# Heck Macrocyclization in Forging Non-Natural Large Rings including Macrocyclic Drugs

**DOI:** 10.3390/ijms24098252

**Published:** 2023-05-04

**Authors:** Jiayou Cai, Bin Sun, Siqi Yu, Han Zhang, Weicheng Zhang

**Affiliations:** The State Key Laboratory of Medicinal Chemical Biology, College of Pharmacy, Tianjin Key Laboratory of Molecular Drug Research, Nankai University, Tianjin 300071, China

**Keywords:** Heck reaction, cross-coupling, ring closure, macrocyclization, macrocyclic drug

## Abstract

The intramolecular Heck reaction is a well-established strategy for natural product total synthesis. When constructing large rings, this reaction is also referred to as Heck macrocyclization, which has proved a viable avenue to access diverse naturally occurring macrocycles. Less noticed but likewise valuable, it has created novel macrocycles of non-natural origin that neither serve as nor derive from natural products. This review presents a systematic account of the title reaction in forging this non-natural subset of large rings, thereby addressing a topic rarely covered in the literature. Walking through two complementary sections, namely (1) drug discovery research and (2) synthetic methodology development, it demonstrates that beyond the well-known domain of natural product synthesis, Heck macrocyclization also plays a remarkable role in forming synthetic macrocycles, in particular macrocyclic drugs.

## 1. Introduction

Heck cross-coupling, alternatively named the Mizoroki–Heck reaction [1], is a time-tested synthetic methodology that has transformed organic chemistry [2,3]. Intramolecularly, the Heck reaction effects cyclic structures ranging from small (*n* = 3–7) through medium (*n* = 8–11) to large (*n* ≥ 12) rings. In contrast to the ample literature addressing Heck-type ring closure for small and medium rings [4,5,6,7,8,9,10], there are only a few works covering the intramolecular Heck reaction for large ring formation [11,12,13], which are unexceptionally dedicated to natural product total synthesis. From time to time, however, this long-neglected transformation, more often termed Heck macrocyclization, has been exploited to prepare synthetic macrocycles including macrocyclic drugs. Having unmatched architecture and functional group disposition, macrocycles constitute a cutting edge of modern drug discovery well poised to engage challenging pharmaceutical targets [14,15,16]. To our surprise, though sporadically mentioned [17,18], the title reaction has hitherto not been scrutinized in the context of making non-natural macrocycles. Accordingly, the present review aims to conduct a systematic survey of this reaction forging diverse synthetic macrocycles beyond natural products. Covering the literature from 1995 to 2022, this review consists of two sections: (1) drug discovery research and (2) synthetic methodology developmentogural products. The first section showcases Heck macrocyclization as employed to build biologically relevant (drug-like) peptidomimetic as well as non-peptidic macrocycles. Of utmost interest is the latest manufacturing route to lorlatinib, a CNS penetrable ALK inhibitor approved for the treatment of lung cancer, which hinges upon a highly efficient intramolecular Heck arylation to close its rigid 13-membered ring. In the second section, attention is paid to the capacity of the Heck reaction to yield an array of unprecedented large rings by virtue of (1) novel allene-containing precursors, (2) sequential multifold couplings, or (3) supramolecular catalysts. Though not immediately translatable to medicinal chemistry, the interesting results compiled in the second part serve to advance our appreciation of the intramolecular Heck reaction and as such will foster its future application in drug synthesis.

## 2. Drug Discovery Research

### 2.1. Peptidomimetic Macrocycles

**Figure 1:**
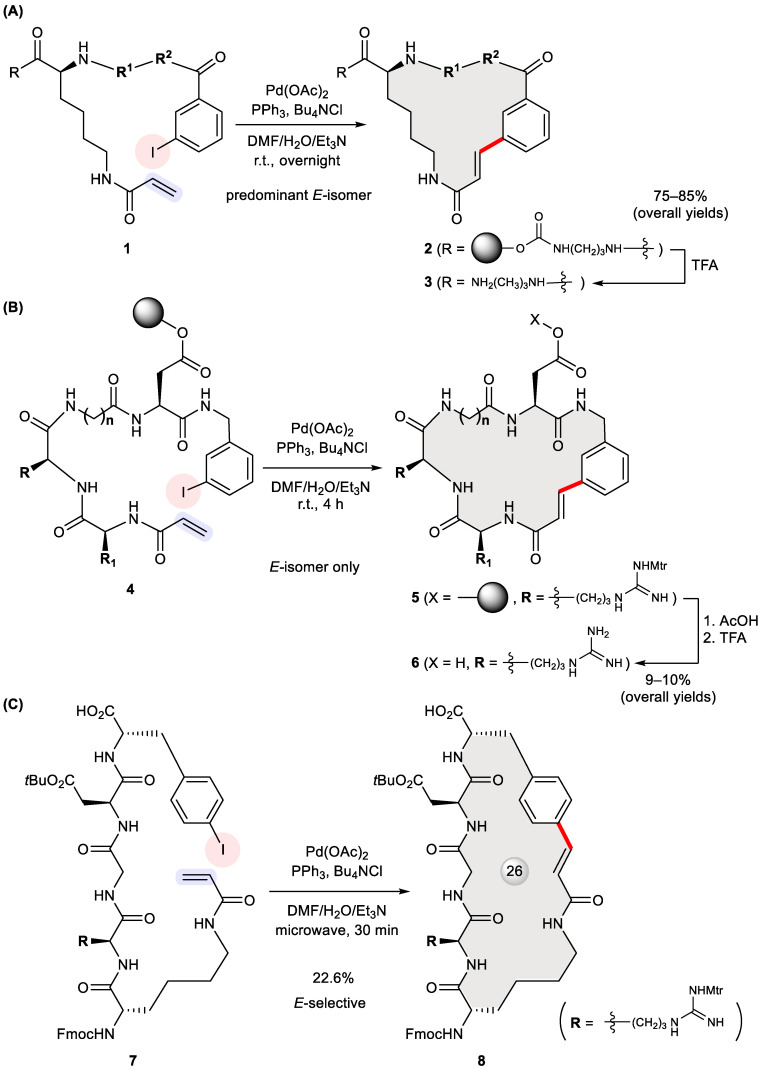
Heck macrocyclization for solid-phase synthesis of cyclic peptidomimetics **3** (**A**), **6** (**B**), and **8** (**C**). (Mtr = 4-methoxy-2,3,6-trimethylbenzenesulfonyl).

Solid-phase synthesis represents a breakthrough technology in organic chemistry, easing product isolation and enabling automated multistep preparation in a combinatorial fashion [19]. Though originally invented for constructing biopolymers, its scope was later broadened to include carbon–carbon bond forming reactions. Hauske et al. depicted the first Pd-mediated macrocyclization in 1995 by means of such a strategy (Figure 1A) [20]. In this pioneering work, a combinatorial library of 15 bifunctional molecules encompassing different amino acid building blocks at R^1^ and R^2^ were grafted to Tenta Gel PHB resin. These supported reactants **1** were treated with Pd(OAc)_2_, PPh_3_, and Bu_4_NCl in a mixed solvent of DMF/H_2_O/Et_3_N at room temperature overnight, followed by TFA-assisted cleavage from the supporting resin for structural analysis. Remarkably, the products **3**, with a variety of ring sizes (20–24 membered) occurring predominantly as *E*-isomers, were recovered in 75–85% overall yields. Two notable features of this system, namely mild cyclization conditions and high yields, may be attributed to the pseudodilution effect [21], a phenomenon referring to the immobilization-induced separation of reactive sites in favor of intramolecular reactions. However, no further biological evaluation of these compounds has been disclosed since.

Solid-phase Heck macrocyclization was also explored in the context of building cyclic tetrapeptides bearing a signature sequence of arginine–glycine–aspartic acid (RGD) [22]. Following an initial proof of concept study [23], Akaji et al. applied the split and mix approach to prepare multicomponent cyclic peptidomimetic libraries **6** varied with ring size (**n**) and substitution at R^1^ via supported Heck macrocyclization of **4** (Figure 1B) [24]. Two combinatorial libraries, each containing 15 compounds, were isolated in 9–10% overall yields after detachment from the resin and deprotection. According to NMR spectroscopy, only *E*-configuration was observed in the nascent alkene. Notably, this heterogeneous cyclization occurred more rapidly than did a corresponding substrate in solution. A preliminary assay found that one purified cyclic RGD derivative (R_1_ = H, *n* = 1 in **6**) from this library selectively inhibited fibrinogen binding to immobilized GPIIb/IIIa with an IC_50_ value of 2 × 10^−5^ M but without inhibitory activity against the vitronectin receptor [25], another member of the integrin family of receptors. In 2006, Byk et al. demonstrated microwave-assisted Heck macrocyclization both on resin and in solution [26]. As an illustration of their approach, an RGD-containing seco precursor **7** prepared via solid-phase synthesis underwent cyclization within 30 min to give biologically relevant macrocycle **8** in a 22.6% yield as an *E*-isomer (Figure 1C). This work shows the potential of microwave-assisted Heck cyclization for preparing conformationally restrained peptidomimetics.

**Figure 2:**
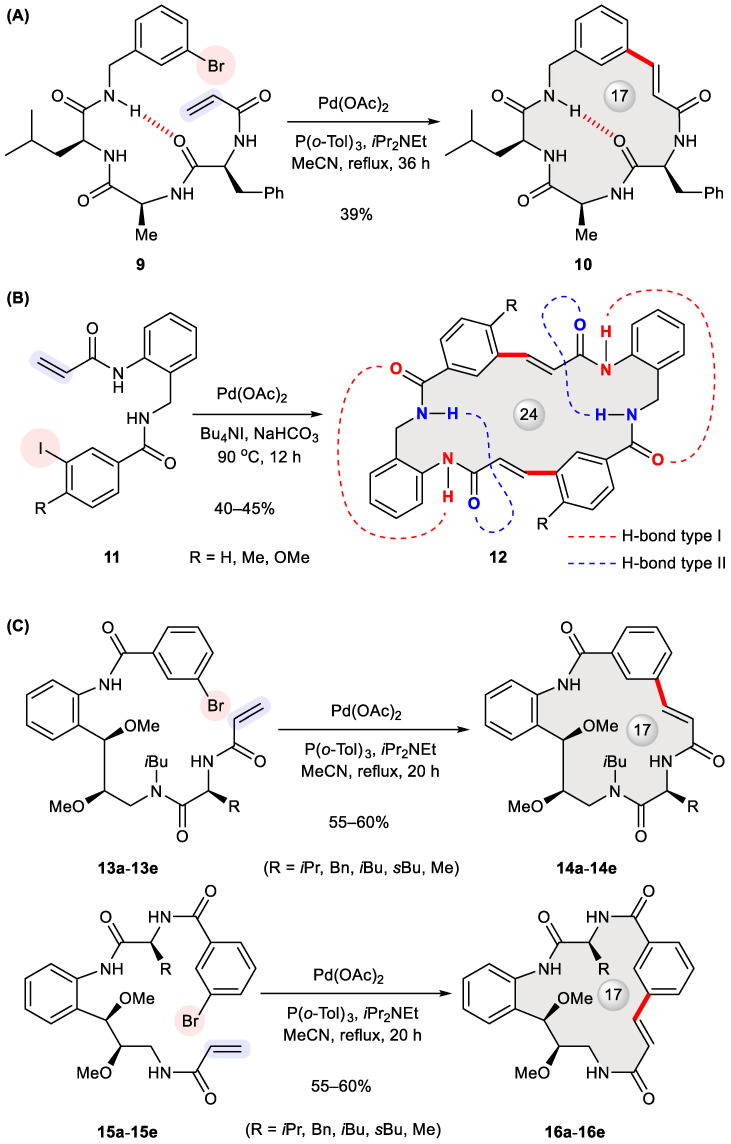
Synthesis of cyclic peptides **10** (**A**), **12** (**B**), **14a**–**14e** and **16a**–**16e** (**C**) via Heck cyclization.

The helix–turn–helix (HTH) motif is instrumental to many DNA-binding proteins such as transcription factors that are capable of recognizing a particular DNA sequence for regulatory purposes [27]. To develop chemical probes gauging DNA–protein interactions, Iqbal et al. designed cyclic peptides bridged by a meta benzene ring such as **10** (Figure 2A) [28]. As the final step of their synthesis, acyclic substrate **9** was treated with Pd(OAc)_2_, tri(*o*-tolyl)phosphine, and diisopropylethylamine in refluxing acetonitrile for 36 h. The product **10** was isolated solely as an *E*-isomer in a 39% yield. Since NMR spectroscopy detected the presence of hydrogen bonds (marked as dashed lines in Figure 2A) in both the linear precursor and its corresponding product, conformational preorganization through such intramolecular hydrogen bonding was believed to promote cyclization.

More recently, Banerji et al. synthesized symmetrical pseudo-turn mimics **12** via double Heck cross-coupling (Figure 2B) [29]. Initially intending to build 12-membered rings, they were unable to obtain any monomeric cyclization product, even under high dilution conditions; rather, dimeric 24-membered macrocycles were produced in 40–45% yields with exclusive *E*-geometry at the newly formed alkenes. NMR and FT-IR spectroscopy proved the presence of intramolecular hydrogen bonds in the precursors **11** as well as the cyclized products **12** (marked in Figure 2B). This stabilizing force was deemed conducive to forming turn-like structures that help with binding to DNA minor grooves [30]. The binding of **11a** (R = H) to DNA minor grooves was confirmed through a variety of experiments including a DAPI displacement assay, mobility shift DNA-binding assay, and melting temperature assay. Based on the fluorescence emission spectra of **5a** at 380 nm in the presence of varying concentrations of CT-DNA, its DNA-binding constant (*K*_A_) was calculated to be 7.89 × 10^4^ M^−1^.

The abundance of bioactive 17-membered natural macrocycles motivated Arya et al. to design two sets of natural product-like compounds **14a**–**14e** and **16a**–**16e** (Figure 2C) [31]. These analogues were prepared in 55–60% yields via Heck macrocyclization of **13a**–**13e** and **15a**–**15e** using Pd(OAc)_2_, P(*o*-tolyl)_3_, and diisopropylethylamine in refluxing acetonitrile. Screening these compounds in various zebrafish assays identified **16a** (R = isopropyl) as a potent antiangiogenic agent with complete inhibition of angiogenesis at 2.5 μM. The fact that its acyclic precursor **15a** was inactive substantiated the importance of a macrocycle-constrained framework.

**Figure 3:**
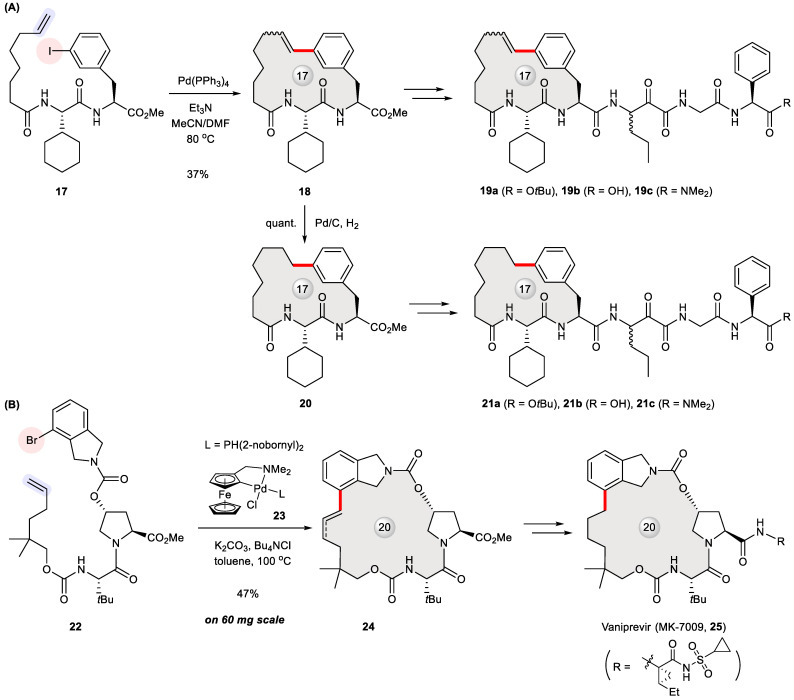
Synthesis of HCV protease inhibitors **19a**–**19c**, **21a**–**21c** (**A**) and vaniprevir (**25**, **B**) via Heck macrocyclization.

Macrocyclization is a popular strategy to create conformationally restrained hepatitis C virus (HCV) non-structural (NS)3/4a protease inhibitors [32], which belong to the group of direct-acting antiviral agents against HCV infection [33]. With a view to enhancing binding affinity and pharmacokinetic properties, Chen et al. designed peptidomimetics **19a**–**19c** and **21a**–**21c** bearing a P2–P3 macrocycle (Figure 3A) [34]. These HCV NS3 protease inhibitors were synthesized via the Heck cyclization of **17** into **18** in a 37% yield as a *Z*/*E*-isomeric mixture. The stapled dipeptide **18** and its hydrogenated intermediate **20** were elaborated at their C-termini to give rise to **19a**–**19c** and **21a**–**21c**, respectively. A bioassay indicated that the presence or absence of an olefinic bond in the macrocyclic tether has a marginal effect on inhibitory activity, while two carboxylic acids **19b** and **21b** were the most potent (Table 1). The conformation of **21b** bound to HCV NS3 protease was further elucidated with X-ray crystallography.

Another pertinent example appeared in 2011 when process chemists at Merck disclosed their first-generation scale-up route to vaniprevir (**25**, MK-7009, shown in Figure 3B) [35], a 20-membered P2–P4 macrocyclic inhibitor of HCV NS3/4a protease [36]. To support clinical development, its practical synthesis was worked out to optimize macrocycle formation. A variety of ring-closing methods were evaluated in terms of robustness and cost-effectiveness, including ring-closing metathesis, Pd-catalyzed macrocyclization, and macrolactamization. Among three Pd-catalyzed cross-couplings tested, the Heck reaction utilizing a hindered ferrocene-based palladacycle **23** stood out with the highest yield (47%) on a 60 mg scale. By contrast, the Suzuki and Sonogashira reactions with their corresponding substrates offered 5% and 35% yields, respectively. Notwithstanding a mixture of configurational and positional isomers, the ring-closed product **24** was hydrogenated and then elaborated to **25**. However, the vaniprevir ring was finally closed via more efficient macrolactamization at a >10 g scale, prior to which intermolecular Heck cross-coupling took place to form the same linkage as created by the previous intramolecular Heck reaction.

**Figure 4:**
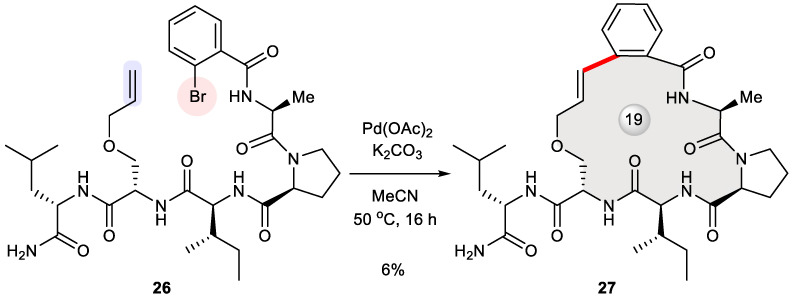
Intramolecular Heck coupling for the synthesis of a stapled pentapeptide **27** as a potential KRAS binder.

Very recently, peptide stapling through macrocyclization reactions [37,38,39] was explored by Spring et al. with a view to discovering a pan-KRAS inhibitor based on the pharmacophore model of a 13-mer peptide binding to KRAS^G12V^. Having identified key interacting residues, they designed a library of smaller peptides whose KRAS-binding conformation as well as drug-likeness is reinforced by means of a one-component stapling strategy [40]. These linear 5- and 6-mer peptides were efficiently assembled via solid-phase peptide synthesis (SPPS) and further subjected to diverse macrocyclization reactions including azide–alkyne cycloaddition, Glaser coupling, ene–yne metathesis, cross-alkene metathesis, Heck cross-coupling, and Sonogashira cross-coupling. While Sonogashira cyclization failed to bring about any observable product, Heck cyclization (unoptimized) smoothly converted pentapeptide **26** into stapled peptide **27** in a modest yield, thus supplying adequate amount of the macrocyclic sample for in vitro biological characterization (Figure 4) [40]. Unfortunately, initial screening for KRAS-binding potency determined its IC_50_ value to be over 100 μM, while more active low-micromolar macrocyclic binders of KRAS were prepared alternatively via Ru-catalyzed azide–alkyne cycloaddition.

### 2.2. Non-Peptidic Macrocycles

**Figure 5:**
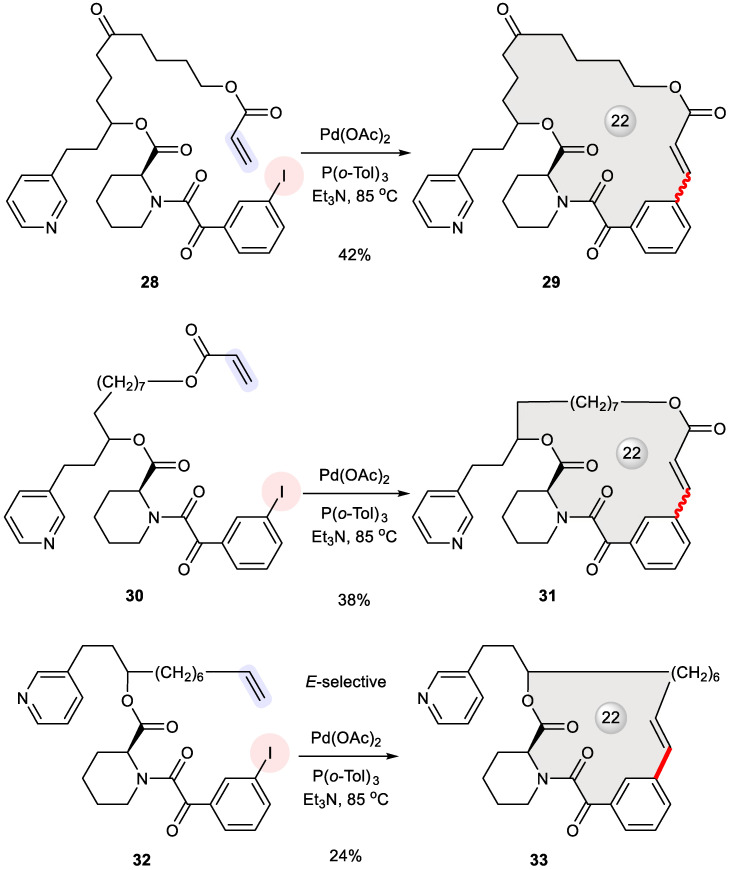
Synthesis of FK506 mimics **29**, **31**, and **33** via Heck macrocyclization.

FK506 is a natural immunosuppressant featuring a 23-membered macrolide, which can be functionally dissected into two domains: one for engaging the FK506 binding protein (FKBP) and the other for downstream signal transduction [41]. To design FK506 mimics with the dual domain concept [42], Stocks et al. reserved a simplified binding domain of the parent macrocycle while replacing its effector domain with hydrocarbon tethers of varying length [43]. For example, macrocycles **29**, **31**, and **33** were prepared from their corresponding acyclic substrates **28**, **30**, and **32** through Heck macrocyclization (Figure 5) using a protocol developed by Gaudin [44]. In the case of **32**, the exclusive formation of *E*-alkenes was observed. Dating back to 1995, this work is among the earliest examples of Heck macrocyclization.

**Figure 6:**
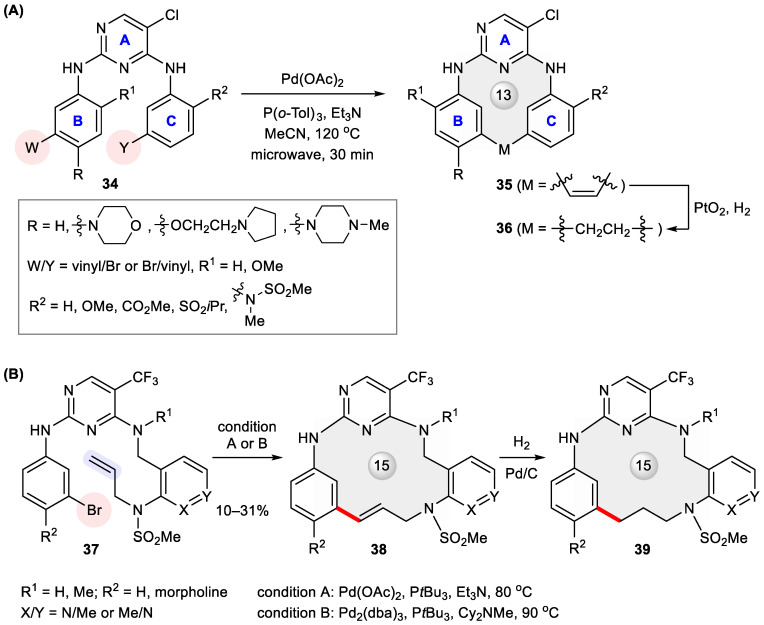
Heck macrocyclization for the preparation of (**A**) ALK inhibitors **35**–**36** and (**B**) Pyk2-selective inhibitors **38**–**39**.

Anaplastic lymphoma kinase (ALK) is a receptor tyrosine kinase implicated in several types of cancer [45]. ALK inhibitors have proved an efficacious modality for targeted cancer therapy [46] since the launch of crizotinib in 2011 for the clinical treatment of ALK-positive non-small cell lung cancer (NSCLC) [47]. Inspired by the inverted U-shaped conformation of a 2,4-diaminopyrimidine (DAP) derivative revealed through X-ray crystallography, Breslin et al. adopted a macrocyclization strategy by designing an array of macrocyclic DAPs **36** capable of enforcing such an active conformation [48]. To access them, acyclic substrates **34** were subjected to microwave-assisted Heck cyclization. The products **35** were obtained in 32–94% yields and further converted to **36** (Figure 6A). Systematic structure–activity relationship (SAR) investigation of thus prepared macrocycles found that sp^2^ hybridization at the restraining two-carbon linchpin (**35**) tends to undermine activity relative to the saturated counterparts (**36**). Among them, **36c** (R = 4-Me-piperazinyl, R^1^ = –OMe, R^2^ = –N(Me)SO_2_Me) exhibited the highest in vitro activity at both enzymatic and cellular levels (IC_50_ = 0.5 nM and 10 nM, respectively), with a desirable kinase selectivity (173-fold) for ALK over the closely homologous insulin receptor (IR) kinase (Table 2). Recently, the same ring scaffolds modified with different phosphine oxides at ring C were disclosed in a patent as potent inhibitors of normal and mutated ALKs [49]. Again, microwave-assisted Heck macrocyclization was invoked to build a heteroaryl tethered 13-membered ring.

The macrocyclization strategy was also applied by Gilead scientists to develop selective inhibitors of proline-rich tyrosine kinase 2 (Pyk2) [50], a potential target for the treatment of invasive cancers [51]. Because Pyk2 shares a similar catalytic domain with focal adhesion kinase (FAK) [52], a challenge is to find a Pyk2-specific inhibitor with low off-target binding to FAK. Starting from PF-562271, a first-in-class dual inhibitor of FAK and Pyk2 [53], ring closure by amidation gave rise to first-generation macrocyclic inhibitors with improved Pyk2 selectivity but unsatisfactory metabolic stability [54]. Introducing a three-carbon linker via Heck macrocyclization led to **38** and **39** (Figure 6B), with not only better stability but also dramatically enhanced Pyk2-binding potency and selectivity compared with their corresponding acyclic precursor **37** (Table 3) [54]. Among these analogues, **38c** displayed the highest stability in the human microsomal stability assay, with a half-life (*t*_1/2_) of 263 min.

Though efficacious against ALK-positive cancers, crizotinib is incapable of blocking mutated ALK, a problem relentlessly haunting the first and second generations of ALK inhibitors. Meanwhile, hardly permeable across the blood–brain barrier (BBB), it cannot control ALK-driven brain metastases that stem from peripheral tumors. To tackle these drawbacks, Pfizer scientists conducted a structure-based drug design that focused on two crucial factors, namely lipophilic efficiency (lipE) and molecular weight (MW) during structural optimization [55]. The former, expressed as pIC_50_ minus log*D* (octanol/buffer distribution coefficient), is a measure of the binding effectiveness of a drug molecule per unit of lipophilicity, helpful to guide the improvement of potency and ADME properties in parallel [56]. Having its root in Lipinski’s rule of five, the latter is negatively correlated with permeability so that a smaller or more compact size is generally preferred. In addition, a substantial challenge is working out a structure efficient at penetrating the BBB to enhance central nervous system (CNS) availability. To this end, an in vitro transwell assay was utilized throughout the drug discovery phase to monitor P-glycoprotein (P-gp) efflux liability in terms of the MDR BA/AB ratio [57]. A high MDR BA/AB ratio (for example, >2.5) indicates significant P-gp efflux and accordingly lack of CNS activity. Through intensive efforts, this drug discovery campaign eventually led to macrocyclic inhibitor lorlatinib (**40**), a third-generation ALK inhibitor and, significantly, the first macrocyclic kinase inhibitor. As shown in Table 4, **40** is highly potent against both wild-type ALK and all known ALK mutants including the gatekeeper L1196M mutant with excellent CNS penetration, which was approved in 2018 for the treatment of NSCLC [58].

**Figure 7:**
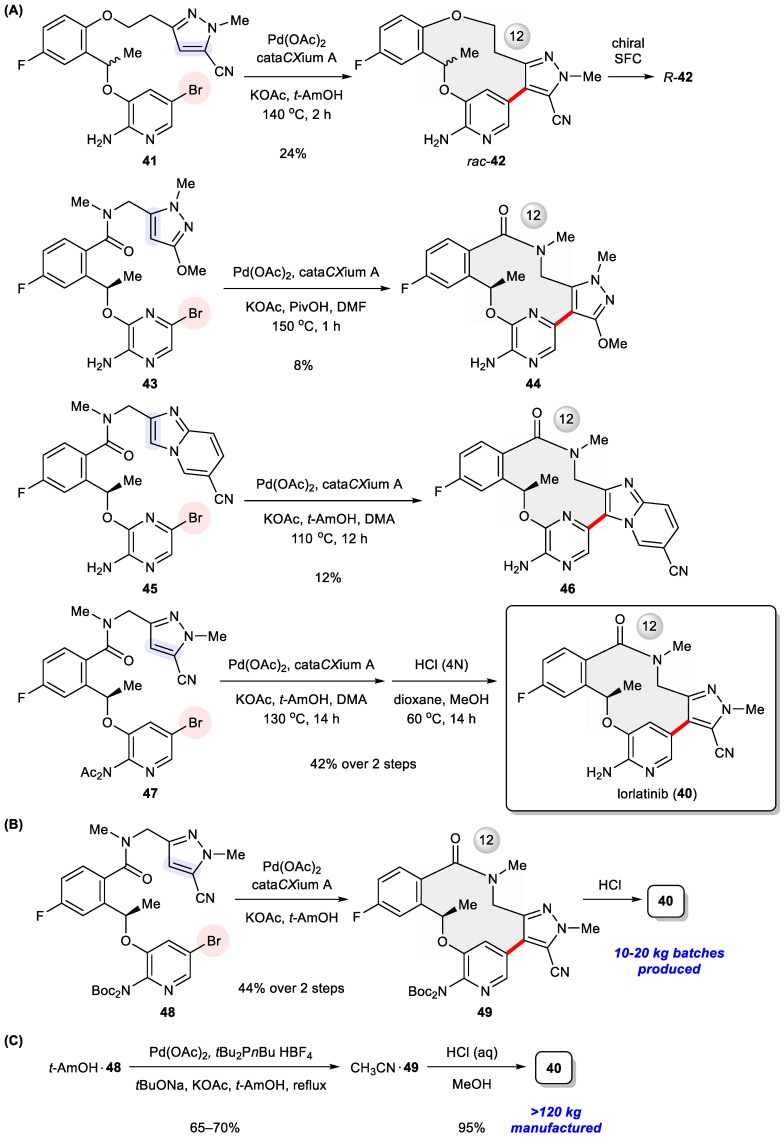
(**A**) Initial medicinal chemistry synthesis of lorlatinib (**40**) and its analogues **42**, **44**, **46** via intramolecular Heck arylation. (**B**) Second-generation and (**C**) commercial-scale synthesis of lorlatinib via intramolecular Heck arylation.

The medicinal chemistry route culminating in **40** required the preparation of diverse 12- to 14-membered macrocycles holding one stereogenic center and three (hetero)aromatic rings. These rigidifying elements make their synthesis a nontrivial task, particularly in view of their close resemblance to synthetically more demanding medium-sized (8- to 11-membered) rings compared with larger (≥15 membered) macrocycles [59]. Intramolecular Heck arylation [6,60], as testified through the successful assembly of numerous 5- and 6-membered rings [61,62,63,64,65,66,67,68,69,70,71], turned out to be indispensable to access macrocyclic (*R*)-**42**, **44**, **46**, and **40** during the initial drug discovery campaign (Figure 7A) [55]. Subsequently, this synthetic approach was implemented to produce radiolabeled isotopologues for positron emission tomography (PET) imaging [72]. Crucial to this ring-closing transformation is the addition of di-1-adamantyl-*n*-butylphosphine (cata*CX*ium^®^A) [73] to promote Pd-catalyzed arylation. In preclinical studies, an exploratory scale-up route was initially reported, relying on amidation to close the macrolactam ring [74]. However, safety concerns over the large-scale use of high-energy condensation reagent HATU prompted Pfizer chemists to work out a second-generation process synthesis through the intermediates **48** and **49** (Figure 7B), in this way delivering 10–20 kg batches of the drug for clinical investigation [75]. To avoid the use of proprietary cata*CX*ium^®^A, an alternative ligand di(*n*-butyl)-*t*-butylphosphine was employed in the form of an air-stable HBF_4_ salt. The endgame of the commercial route features an efficient intramolecular Heck arylation of crystalline **48** *t*-amyl alcohol to yield another crystalline solvate, **49** acetonitrile, in 65–70% yields, followed by acidic deprotection so as to manufacture the API at a >120 kg scale for the time being (Figure 7C) [75]. It is worth noting that intramolecular Suzuki coupling had been evaluated in parallel throughout the process optimization study, but poor yields were achieved even after extensive screening of multiple reaction parameters and, accordingly, its applicability was ruled out.

### 2.3. Natural Product Analogues

**Figure 8:**
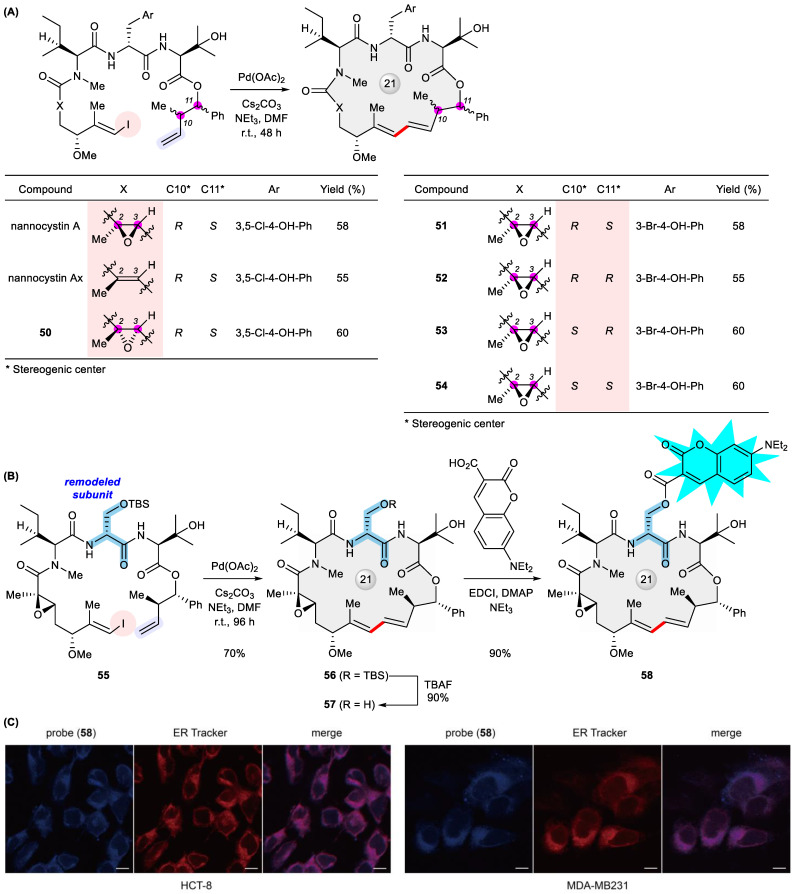
(**A**) Synthesis of nannocystin A and analogues **50**–**54** permutated stereochemically along the macrocyclic backbone via Heck macrocyclization. (**B**) Synthesis of a diversity-conferring nannocystin intermediate **57** via Heck macrocyclization and elaboration into a macrocyclic fluorescent probe **58**. (**C**) Confocal fluorescence images of cancer cells co-stained with ER-tracker and **58** (scale bars, 10 μm). Reproduced from ref. [90] with permission from the Royal Society of Chemistry.

In a broad sense, natural product analogues should contain those initially targeted for total synthesis but only found later to be misassigned structures, a serendipitous twist alluding to the charm of natural product research. Pertaining to the title reaction, the synthesized nominal structures of diazonamide A [76], kulkenon [77], the aglycone of mandelalide A [78], and maltepolide C [79] belong to this category. More often though, analogues of structurally validated natural products are prepared deliberately, rather than unexpectedly, in order to explore biologically relevant chemical space, wherein Heck macrocyclization again plays a substantial role. Earlier examples include conformationally restricted taxoids [80], side chain derivatives of mandelalide A [81], and stereodivergent solomonamides [82]. Recently, our total synthesis of highly antiproliferative nannocystin A through Heck macrocyclization [83,84] secured subsequent SAR investigations by facilely preparing dozens of non-natural analogues including **50**–**54** that deviate from the natural lead either stereochemically, along the macrocycle backbone, or at the peripheral substituent (Figure 8A) [85,86,87,88]. Aiming at site-directed late-stage diversification for quickly exploring chemical space around the nannocystin framework [89], we next remodeled its macrocycle composition in which a homochiral serine (highlighted in the structure) has been substituted for the innate D-serine to give the macrocyclic alcohol **57**. To our satisfaction, the precursor **55** underwent smooth ring closure in a 70% yield under Heck coupling conditions, TBS deprotection then furnishing **57** ready for divergent post-macrocyclization esterification (Figure 8B) [90]. Although nannocystin A was shown to be a specific inhibitor of eukaryotic elongation factor 1A (eEF1A) [91], its exact anticancer mode of action awaits further elucidation [92]. Informed by thus obtained SAR and pursuing a nannocystin-based fluorescent probe [93], we designed and synthesized a coumarin conjugate **58** with good cell permeability. It was observed by means of confocal fluorescent microscopy that this probe is localized predominantly to the endoplasmic reticulum (Figure 8C), most likely acting upon its target eEF1A at the ER-bound ribosome [90]. Interestingly, our result is in good agreement with a recent work that visualized eEF1A associated with the ribosome on the ER membrane at molecular resolution by the use of cryo-electron tomography [94], thereby shedding light on the intracellular mode of action of nannocystins.

## 3. Synthetic Methodology Development

### 3.1. Allenic Precursors

**Figure 9:**
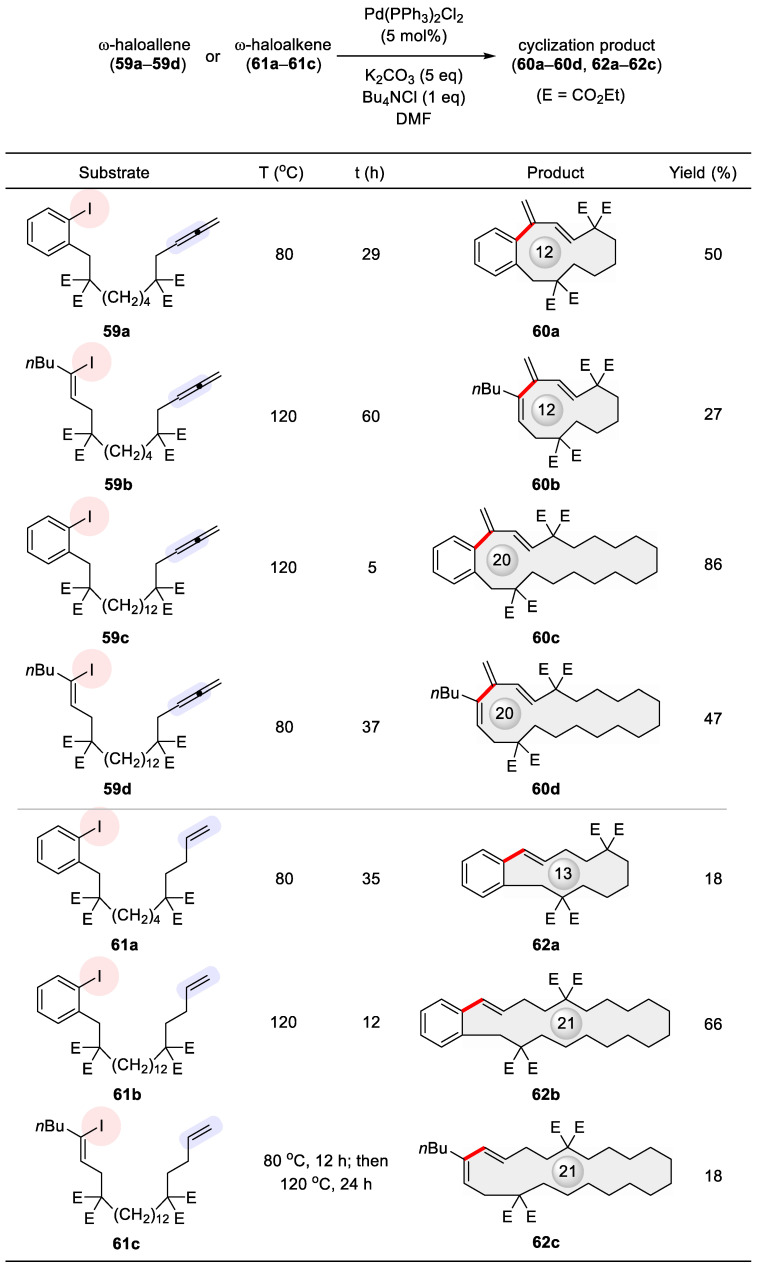
Heck macrocyclization using allene **59a**–**59d** or alkene **61a**–**61c** as the alkenyl coupling partner.

An interesting Heck-type cyclic carbopalladation was reported by Ma and Negishi in 1995, employing allenes as the alkenyl coupling partner, thereby producing carbocycles of varying sizes including macrocycles [95]. This work was based on their earlier discovery of facile access to 7- and 8-membered rings from allene-containing organohalides via Pd catalysis [96]. Substrates pertaining to the subject of this review are given in Figure 9, including ω-iodoallenes **59a**–**59d** and ω-iodoalkenes **61a**–**61c** highlighted at their alkenyl functionalities. Aside from catalytic amount (5 mol%) of Pd(PPh_3_)_2_Cl_2_ and five equivalents of K_2_CO_3_, critical to their ring closure are (1) the addition of the phase transfer agent Bu_4_NCl as pioneered by Jeffery [97] and (2) executing the reaction at reduced concentrations. Despite three possible pathways for carbon–carbon bond formation, the actual cyclization took place invariably at the central allenic carbon with exclusive formation of an exo alkene. The 12- and 20-membered rings **60a**–**60d** were prepared from their allenic precursors **59a**–**59d** in higher yields than the 13- and 21-membered endo macrocycles from their corresponding ω-iodoalkenes (**59a** vs. **61a**, **59c** vs. **61b**, **59d** vs. **61c**, Figure 9). Such a superior performance may originate from the cumulated double bonds of allenes, which have gained increasing popularity in organic synthesis [98,99,100,101,102].

**Figure 10:**
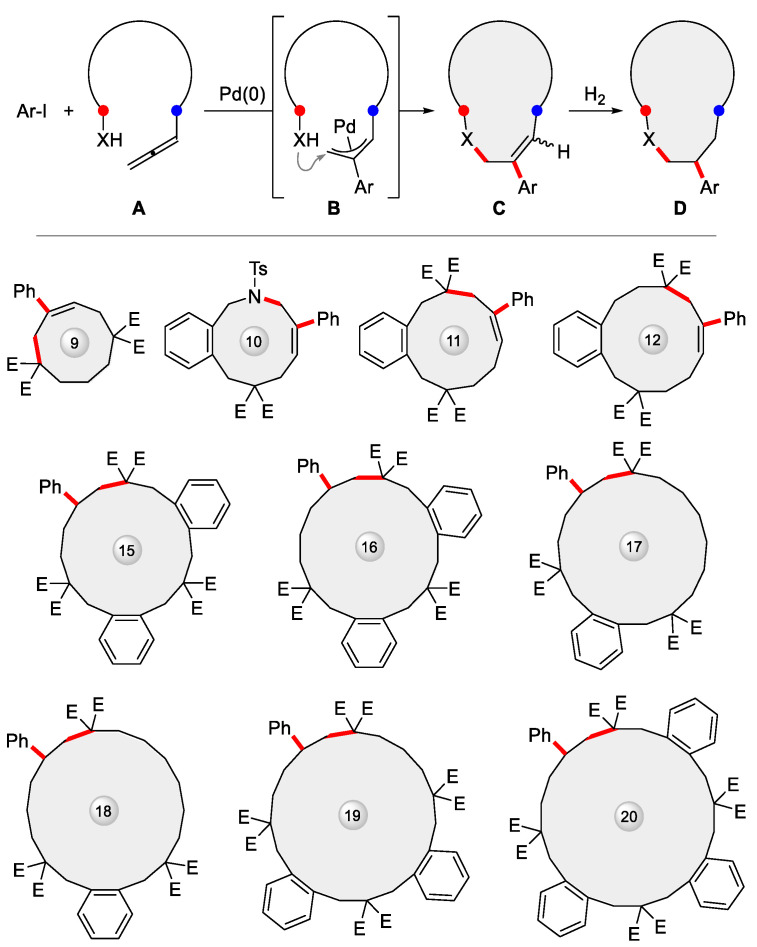
Pd-catalyzed intermolecular cyclization between Ar-I and allenes carrying a remote tethered nucleophile X brought about diverse medium and large rings (E = CO_2_Et). **A**: nucleophile-tethered allene; **B**: reaction intermediate; **C**: cyclized product; **D**: further hydrogenated product.

More recently, this methodology was upgraded to embrace intermolecular cyclization between organoiodides and allenes (Figure 10 top), where the allene coupling partner **A** was equipped distally with an extra nucleophile X so as to self-trap the transient allylpalladium species **B** generated from initial addition of Ar-Pd-I, resulting in **C** with high regio- and *E*/*Z* stereoselectivity, finally yielding the saturated ring **D** via Pd/C hydrogenation [103,104]. A variety of unprecedented 9–20-membered rings were prepared via this strategy, as showcased in Figure 10 (bottom).

### 3.2. Single, Double, or Multifold Heck Reactions

**Figure 11:**
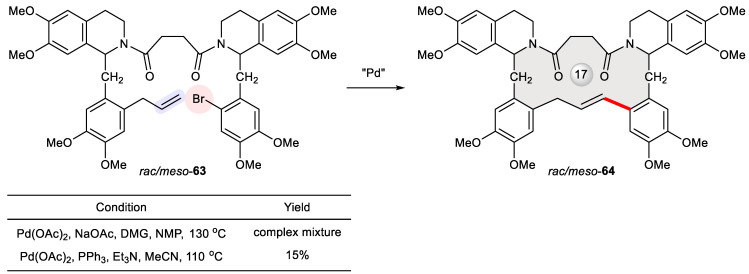
Synthesis of macrocyclic laudanosine dimer **64** via Heck cyclization of *rac*/*meso*-**63**.

In exploring novel analogues of bisbenzylisoquinoline alkaloids [105], Pyne et al. synthesized laudanosine dimers bound with carbon tethers [106]. Since laudanosine is an active metabolite of the neuromuscular-blocking drugs atracurium and cisatracurium [107], its dimer may have interesting properties. One compound they obtained is macrocyclic **64,** deriving from the intramolecular Heck reaction of the acyclic substrate **63**. Conventional Heck reaction conditions using Pd(OAc)_2_, PPh_3_, and Et_3_N at 110 °C delivered **64** in a 15% yield, whereas the optimal conditions for the intermolecular Heck reaction of other laudanosine analogues utilizing Pd(OAc)_2_, NaOAc, *N*,*N*-dimethylglycine, and NMP at 130 °C paradoxically resulted in a complex mixture (Figure 11).

**Figure 12:**
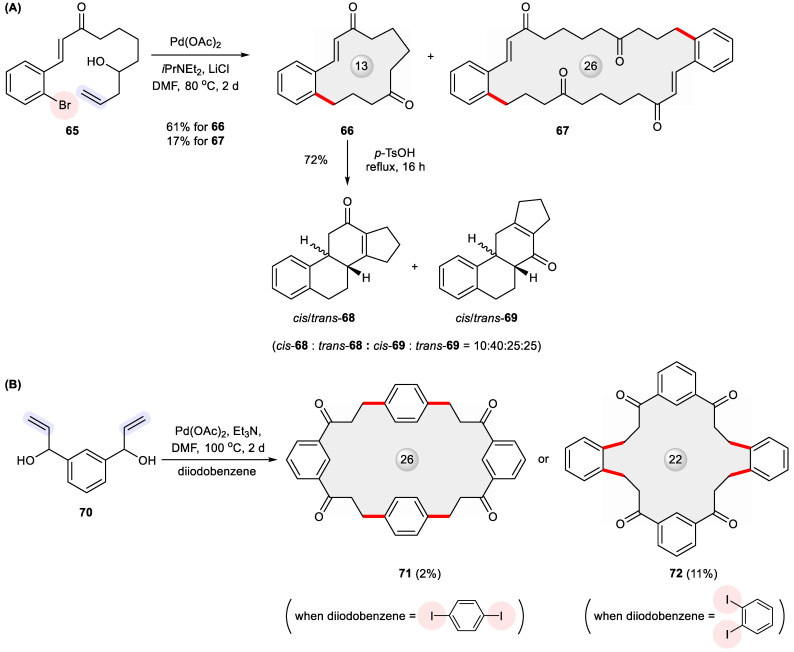
Heck macrocyclization for constructing (**A**) steroid frameworks **68**–**69** from homoallylic alcohol **65**, as well as (**B**) ketonic macrocycles **71** and **72** from bisallylic alcohol **70**.

Due to the reversibility of β-hydride elimination, the Heck cross-coupling of allylic and homoallylic alcohols renders carbonyl products through double bond migration [108]. Coupled with other transformations, this reaction can initiate domino processes that give annulated ring systems. Pursuing this goal [109,110,111,112,113], Dyker et al. devised a double transannular cyclization strategy to access the tetracyclic steroid skeleton in the form of isomeric *cis*/*trans*-**68** and *cis*/*trans*-**69**, which relied on the intramolecular Heck cyclization of allylic alcohol **65** to form the precursor macrocycle **66** (Figure 12) [114]. In addition to **66**, a 26-membered macrocycle **67** was isolated in an unneglectable yield of 17%. Intrigued by this finding, the authors performed a follow-up study to show the opportunity of creating *C*_2v_-symmetric macrocycles **71** and **72** through a fourfold Heck reaction with *p*-diiodobenzene and *m*-diiodobenzene, respectively [115].

**Figure 13:**
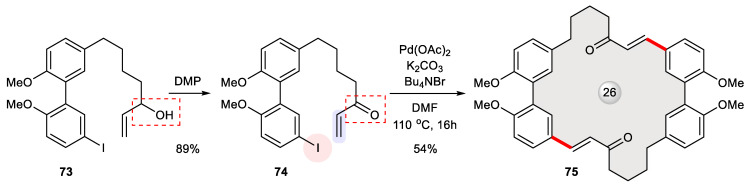
Double Heck cross-coupling of **74** leading to 26-membered cyclic dimer **75**.

The double Heck cyclization approach was likewise investigated by Harrowven et al. for macrocycle synthesis [116]. Because the formation of a biphenyl-embedded 13-membered ring from **73** is likely to experience significant strain, as encountered in an independent study before [117], the authors envisioned that such an energetically disfavored process would be surpassed by the closure of a relaxed dimeric 26-membered ring (Figure 13). Nevertheless, the initial precursor **73** failed to cyclize under Pd(0) catalysis; a mixture of polar by-products were detected instead, likely as a result of competitive polymerization. The impasse was overcome by oxidizing its allylic alcohol (highlighted in Figure 13) with Dess–Martin periodinane (DMP). The resulting α,β-unsaturated ketone **74** gratifyingly boosted the anticipated intermolecular–intramolecular Heck couplings, affording **75** in a 54% yield. Echoing the preference for dimer formation, recently, an unexpected 26-membered macrocyclic dimer was observed via RCM in an attempted total synthesis of myricanol wherein no mono-cyclization occurred to give a strained 13-membered ring [118].

**Figure 14:**
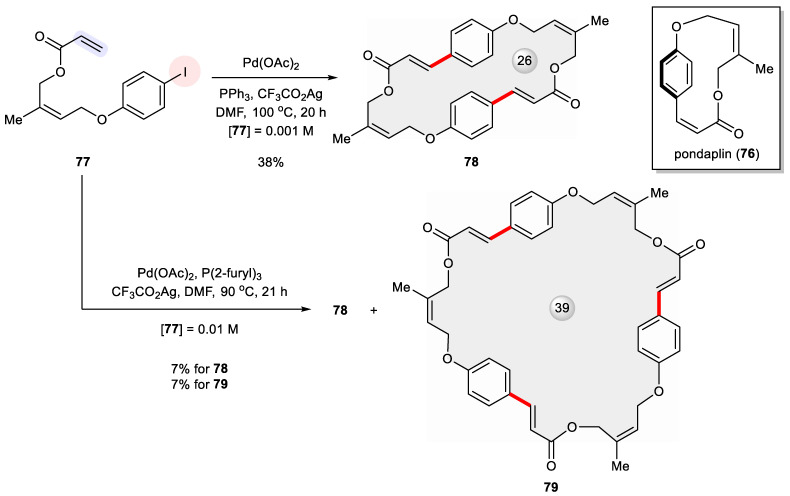
Attempted total synthesis of pondaplin (**76**) via Heck cyclization of **77** unexpectedly led to its dimeric and trimeric macrocycles **78** and **79**.

Pondaplin (**76**) is a strained 13-membered macrocycle because of its rigid 1,4-benzenoid linkage and two built-in *Z*-alkenes [119]. In an effort to synthesize **76**, Joullie et al. explored the intramolecular Heck reaction of the seco precursor **77** but without yielding the target molecule under various conditions (Figure 14) [120]. A serendipitous result from their trials was the formation of the pondaplin dimer **78** in a 38% yield under high dilution conditions (0.001 M). Clearly, high strain energy accrued along the self cross-coupling pathway and as such defied ring closure. As a result, the sequential intermolecular–intramolecular process came into play and generated the dimeric macrocycle. At a 10-fold increased concentration, that is, 0.01 M of **77**, a head-to-tail cyclized trimer **79** (7% yield) was isolated along with **78** (7% yield).

**Figure 15:**
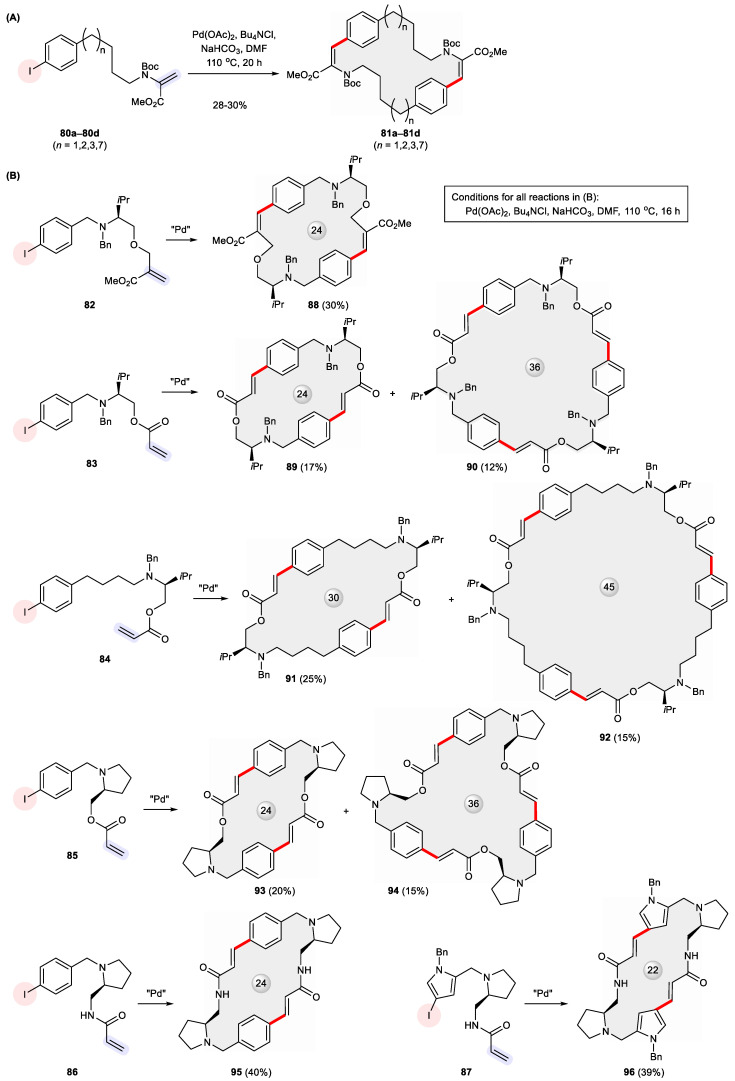
Formation of (**A**) benzene-bridged cyclophanes **81a**–**81d** bearing (*Z*)-dehydrophenylalanine substructures via double Heck cross-coupling and (**B**) chiral dimeric and/or trimeric macrocycles **88**–**96** via multifold Heck reaction.

The versatility of multifold Heck-type cross-coupling [121] was demonstrated by Gibson et al. for rapidly preparing a collection of dimeric and trimeric macrocycles from simple starting materials. Both achiral [122,123] and chiral [124,125,126] macrocycles were produced via this strategy. As shown in Figure 15A, achiral cyclophanes possessing two (*Z*)-dehydrophenylalanine subunits such as **81a**–**81d** arise from the Heck-type head-to-tail dimerization of the corresponding dehydroalanine derivatives **80a**–**80d**, to which *p*-iodobenzene is attached via a hydrocarbon spacer of different lengths [123]. On the other hand, a variety of bifunctional ω-iodo-1-alkenes **82**–**87** derived from (*S*)-valinol (for **82**–**84**) or (*S*)-prolinol (for **85**–**87**) underwent double and/or triple Heck cross-coupling, giving rise to chiral macrocycles of varying ring sizes, as depicted in Figure 15B [124,125,126].

### 3.3. Supramolecular Catalysts

**Figure 16:**
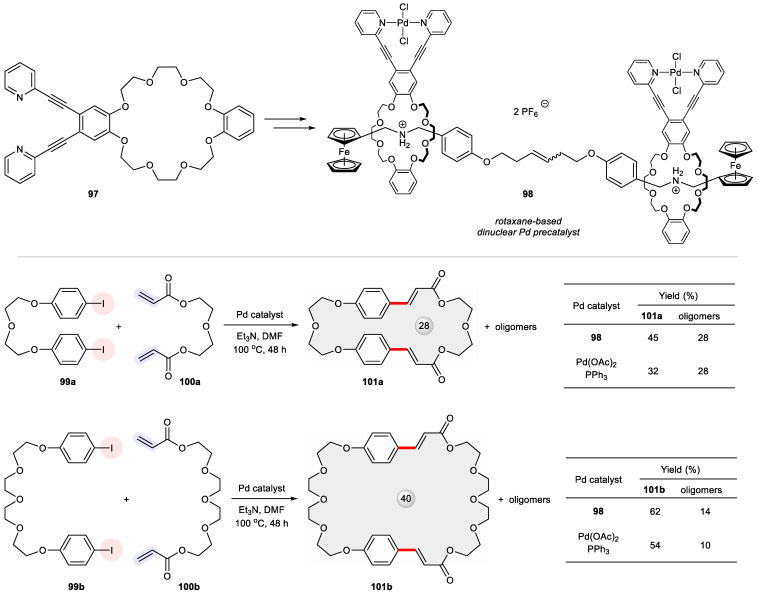
Rotaxane-based dinuclear Pd precatalyst **98** for Heck-type macrocyclization between **99** and **100**.

An intriguing case was made by the use of a dinuclear Pd precatalyst **98** mounted onto a rotaxane platform (Figure 16) [127]. This mechanically interlocked supramolecular catalyst was derived from a bidentate *N*,*N* ligand **97** containing a crown ether motif so that two Pd-complexed macrocycles could be threaded through a confining α,ω-bisferrocenyl shaft. Its catalytic performance was compared with the standard Pd(OAc)_2_/PPh_3_ system in the Heck cross-coupling of two pairs of bifunctional substrates, namely (1) **99a**, **100a** and (2) **99b**, **100b**. While both reactions suffered from competing oligomerization, the rotaxane-based catalyst **98** produced higher yields of the macrocycles **101a** and **101b** relative to oligomers than the discrete Pd species.

## 4. Conclusions and Future Perspectives

**Figure 17:**
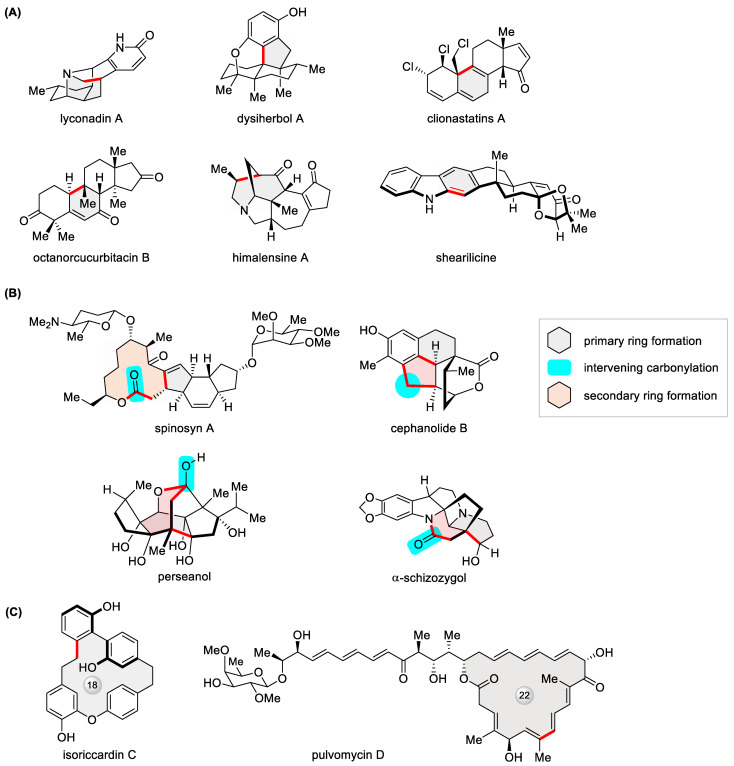
Recent examples of polycyclic natural products constructed via (**A**) intramolecular Heck reaction and (**B**) Heck-type carbonylative tandem cyclization, as well as (**C**) macrocyclic natural products built via Heck macrocyclization.

In contrast to alternative Pd-catalyzed macrocyclization reactions [11], an appealing facet of the Heck reaction lies in the fact that there is no prerequisite for a heteroatom-functionalized alkene coupling partner. Hence, it is an apt embodiment of the KISS (keep it simple …) principle with regard to atom economy. The absence of a directing/activating group, such as a boronic acid moiety, which defines the Suzuki reaction, is prone to engendering ambiguous regio- and stereoselectivity, an issue often challenging intermolecular Heck cross-coupling. Fortunately, such a complication is less seen in intramolecular Heck cyclization. In truth, its relative lack of certainty or predictability compared with other well-practiced cross-coupling processes happens to call for a creative mindset in synthetic design, as elegantly exemplified by the recent total synthesis of polycyclic natural products such as lyconadin A [128], dysiherbol A [129], clionastatins A [130], octanorcucurbitacin B [131], himalensine A [132], and shearilicine [133] (Figure 17A). When coupled to carbonylation with the one-carbon feedstock CO, the intramolecular Heck reaction offers the opportunity to generate two consecutive rings in a one-pot cascade [134]. As illustrated in Figure 17B, Pd-catalyzed carbonylative Heck-type macrolactonization, C-H functionalization, lactonization, and lactamization have inspiringly led to the total synthesis of spinosyn A [135], cephanolides B [136], perseanol [137], and α-schizozygol [138], respectively. With regard to large rings, shortly after a 2021 review [12], more progress was made in the total synthesis of isoriccardin C [139], pulvomycin D (Figure 17C) [140], and the (2*E*) isomer of macrolactin 3 [141].

The theme of the present review, on the other hand, is to raise awareness of the fact that this reaction is likewise useful in forging synthetic macrocycles, especially macrocyclic drugs. Although first reported in the early 1980s [142], it was only more than 10 years later, that is, in 1995, that three independent studies led by Ma and Negishi [95], Hauske [20], and Stocks [43] attested the utility of the intramolecular Heck reaction in generating innovative synthetic macrocycles. These achievements, in turn, could have inspired broader exploration of Heck macrocyclization in natural product synthesis, as initiated by Harran’s landmark synthesis of the originally proposed structure of diazonamide A [76,143,144]. Aside from being investigated as a synthetic method, this reaction has made prominent contributions to macrocyclic drugs including stapled peptides as well as non-peptidic kinase inhibitors, as evidenced by the lab-scale and commercial synthesis of lorlatinib, an approved latest-generation broad-spectrum ALK inhibitor bearing a rigid 13-membered biheteroaryl macrolactam ring. In this regard, Heck cross-coupling proves a viable option in the arsenal of available macrocyclization reactions to enable macrocycle-based drug design, a frontier of modern drug discovery [145,146,147,148,149,150,151,152].

Thus far, both phosphine-assisted and phosphine-free catalytic systems have found wide applications in Heck macrocyclization (Table 5). Of note, the former proceeds at elevated temperatures typical of a routine inter- or intramolecular Heck reaction, often adopted to prepare non-natural macrocycles, whereas the latter involves a much milder condition amenable to the total synthesis of natural macrocycles and their analogues. These contrasting reaction settings betoken fundamentally different emphases in making natural products and designer macrocycles. To access a non-natural macrocycle, particularly a macrocyclic drug under development, efficiency is a pivotal factor, so drastic conditions are required to drive the completion of large ring formation. When it comes to constructing a natural macrocycle, however, caution must be exercised to not spoil its rich functional groups. Therefore, it is reasonable to effect large ring closure at or close to room temperature to reduce side reactions. It is evident that no matter which method is used, the yield of Heck macrocyclization remains suboptimal, ranging from moderate to good yet seldom reaching up to 90% (Table 5). Consistent with this observation, Knapp and Hanke et al. recently assessed the efficiency of various macrocyclization reactions yielding macrocyclic kinase inhibitors reported over the past 15 years [59] in terms of the Emac index [153]. Clearly, there is much room to improve the productivity of Heck macrocyclization when compared with conventional macrocyclization reactions such as macrolactonization and macrolactamization. Looking forward, this would likely be accomplished through not only extensive optimization of reaction parameters (ligand, additive, solvent, temperature, etc.) during scale-up, but more decisively, in the long run, the advent of more capable catalytic systems. Moreover, it is the existence of stable, long-acting Pd species that could sustain efficient large ring formation in a highly diluted medium. As the macrocyclization strategy extends outside the sphere of kinase inhibitors to harness mechanistically diverse therapeutic agents, Heck macrocyclization will reveal its value in due course.

## Figures and Tables

**Table 1:** Inhibitory activity of macrocyclic peptidomimetics **19a**–**19c** and **21a**–**21c** against HCV NS3 protease.

Compound	IC_50_ (μM)	Compound	IC_50_ (μM)
**19a**	1.2	**21a**	2.3
**19b**	0.084	**21b**	0.066
**19c**	0.12	**21c**	0.11

**Table 2:** SAR of DAP-containing macrocyclic drugs **35a**–**35c** and **36a**–**36c**.

Compound ^a^	M	R^1^	R^2^	ALK IC_50_ (nM)	Selectivity ^b^
**35a**	(*Z*)-(CH=CH)	H	H	392 ± 149	4
**35b**	(*Z*)-(CH=CH)	H	OMe	259 ± 74	>11
**35c**	(*Z*)-(CH=CH)	OMe	N(Me)SO_2_Me	7.0 ± 0.8	>140
**36a**	CH_2_CH_2_	H	H	92 ± 10	2.8
**36b**	CH_2_CH_2_	H	OMe	3.1 ± 0.7	67
**36c**	CH_2_CH_2_	OMe	N(Me)SO_2_Me	0.51 ± 0.02	173

^a^ Structures shown in Figure 6A, R = 4-Me-piperazinyl for all compounds. ^b^ Selectivity = (IR IC_50_)/(ALK IC_50_).

**Table 3:** SAR of Pyk2-targeting macrocyclic inhibitors **38a**–**38c** and **39a**–**39b** and their acyclic precursors **37a**–**37c** (structures shown in Figure 6B).

**Compound**	**R^1^**	**R^2^**	**X**	**Y**	**Pyk2 IC_50_ (** **nM)**	**FAK IC_50_ (** **nM)**
**37a**	H	H	N	CH	122	0.51
**38a**	H	H	N	CH	2.60	10.2
**39a**	H	H	N	CH	0.67	1.26
**37b**	H	morpholine	N	CH	19.5	0.51
**38b**	H	morpholine	N	CH	0.84	4.34
**39b**	H	morpholine	N	CH	1.31	3.21
**37c**	Me	morpholine	CH	N	6625	7496
**38c**	Me	morpholine	CH	N	2.70	14.0

**Table 4:**
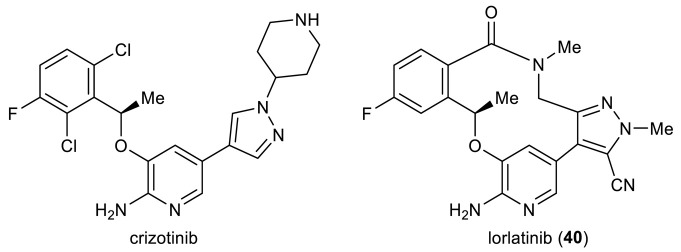
Potency and key physicochemical properties of crizotinib and lorlatinib (**40**).

Compound	ALK Cell IC_50_ (nM)	ALK-L1196M Cell IC_50_ (nM)	log *D*	LipE	MDR BA/AB Ratio
crizotinib	80	843	2.0	4.1	44.5
lorlatinib (**40**)	1.3	21	2.3	5.4	1.5

**Table 5:** Two types of Pd-based catalytic systems for Heck macrocyclization and application examples thereof.

Classification	Typical Reaction Condition	Examples	Yield (%)	Ref.
Phosphine-assisted	Pd(OAc)_2_, P(*o*-Tol)_3_, *i*Pr_2_NEt, MeCN, heating	Cyclic peptide **10**	39	[28]
		Cyclic peptide **14**, **16**	55–60	[31]
	Pd(OAc)_2_, P(*o*-Tol)_3_, NEt_3_, MeCN, microwave heating	ALK inhibitor **35**	32–94	[48]
		ALK inhibitor (patented)	39	[49]
	Pd(OAc)_2_, cata*C*Xium A or *t*Bu_2_P*n*Bu·HBF_4_, KOAc, *t*-AmOH, heating	Lorlatinib (**40**) and analogues	8–36	[55,75]
		Lorlatinib manufacture	65–70	[75]
Phosphine-free	Pd(OAc)_2_, *n*Bu_4_NCl, K_2_CO_3_, DMF, heating	Etnangien	47	[154]
		Pestalotioprolide G	23	[155]
		Biselyngbyolide B	58	[156]
	Pd(OAc)_2_, Cs_2_CO_3_, NEt_3_, DMF, r.t.	Palmerolide A	81	[157]
		Mandelalide A and analogues	70–80	[78,81]
		Nannocystin A and analogues	55–70	[83,85,86,87,88,90]
		(2*E*)-Macrolactin 3	71	[141]
